# Rice volatiles lure gravid malaria mosquitoes, *Anopheles arabiensis*

**DOI:** 10.1038/srep37930

**Published:** 2016-11-30

**Authors:** Betelehem Wondwosen, Göran Birgersson, Emiru Seyoum, Habte Tekie, Baldwyn Torto, Ulrike Fillinger, Sharon R. Hill, Rickard Ignell

**Affiliations:** 1 Department of Zoological Sciences, Addis Ababa University, P. O. Box1176, Addis Ababa, Ethiopia; 2 Unit of Chemical Ecology, Department of Plant Protection Biology, Swedish University of Agricultural Sciences, P. O. Box 102, Sundsvägen 14, 230 53 Alnarp, Sweden; 3 Behavioural and Chemical Ecology Department, International Centre of Insect Physiology and Ecology, P. O. Box 30772, Nairobi 00100, Kenya; 4 Disease Control Department, London School of Hygiene and Tropical Medicine, London WC1E 7HT, UK

## Abstract

Mosquito oviposition site selection is essential for vector population dynamics and malaria epidemiology. Irrigated rice cultivations provide ideal larval habitats for malaria mosquitoes, which has resulted in increased prevalence of the malaria vector, *Anopheles arabiensis*, in sub-Saharan Africa. The nature and origin of the cues regulating this behaviour are only now being elucidated. We show that gravid *Anopheles arabiensis* are attracted and oviposit in response to the odour present in the air surrounding rice. Furthermore, we identify a synthetic rice odour blend, using electrophysiological and chemical analyses, which elicits attraction and oviposition in laboratory assays, as well as attraction of free-flying gravid mosquitoes under semi-field conditions. This research highlights the intimate link between malaria vectors and agriculture. The identified volatile cues provide important substrates for the development of novel and cost-effective control measures that target female malaria mosquitoes, irrespective of indoor or outdoor feeding and resting patterns.

Oviposition site selection by malaria mosquitoes is a behaviour that needs to be exploited in future control strategies. Although existing indoor control methods, including indoor residual spray (IRS) and long lasting insecticidal nets (LLINs), have been effective in reducing indoor malaria transmission^1^, we are now seeing a reduction in that efficacy^2^, likely due to the emergence of insecticide resistance^3^,^4^,^5^, the resurgence of vectors^6^,^7^, and the shift from indoor to outdoor vector behaviour^7^,^8^,^9^,^10^ leading to a proportional increase in outdoor malaria transmission^11^,^12^,^13^,^14^. In many areas, this change is associated with one of the primary malaria vectors, *Anopheles arabiensis*^6^. Besides larval control, which has largely been disbanded in favour of IRS and LLINs^15^ in contemporary sub-Saharan Africa, there are no effective control strategies that target malaria mosquito populations outdoors. Gravid malaria mosquitoes, irrespective of indoor or outdoor feeding patterns, must seek for suitable aquatic habitats to lay their eggs. Identifying and manipulating cues from such habitats could provide important insights toward novel mosquito control tools.

Selection of oviposition sites for vectors of malaria is dependent on a number of physical and chemical factors. Gravid malaria mosquitoes are attracted to water bodies by water vapour^16^, as well as olfactory cues from conspecific larvae^17^,^18^, microorganisms^19^ and soil^20^ associated with the sites. To date, the only compound confirmed as an oviposition attractant for malaria mosquitoes is cedrol, a soil-associated compound^20^. While habitat cues associated with the sites potentially attract mosquitoes over a longer distance, these have not been fully explored. Emergent and other vegetation may provide such potential cues, as grasses and other short vegetation often emerge from and surround natural larval habitats of malaria mosquitoes^21^,^22^.

Governmental and non-governmental initiatives are systematically increasing the regions under irrigated cultivation throughout sub-Saharan Africa^23^. Although irrigated cultivations of cereal crops, including rice, maize and sugarcane, provide food security, they also provide ideal larval habitats for malaria mosquitoes^24^,^25^. This generally creates a spatial link between the vector and the people, thereby increasing the risk of malaria transmission. The cereal crops provide the larvae with shelter from abiotic and biotic threats, as well as nutrients directly from shed pollen and indirectly from accumulated detritus and associated microorganisms^25^,^26^. Irrigated rice agro-ecosystems, in particular, can create permanent mosquito larval habitats^27^, thereby increasing the abundance of malaria vectors compared with other cultivated cereals^28^,^29^,^30^. Moreover, the agronomic activities in rice, including the addition of fertilizers and insecticides^31^,^32^, can also affect larval density by increasing available nutrients^33^ and creating predator-free habitats^34^, respectively. Thus, female mosquitoes that select breeding sites within irrigated rice and other cereal cultivations have the potential to increase their own fitness by providing their offspring with selective advantages. We show that gravid *An. arabiensis*, one of the predominant mosquito species in rice cultivation^35^,^36^, are attracted to the odour of rice, irrespective of the phenological stage of the plant, and identify a synthetic blend of rice-volatiles that acts as a long-range attractant and elicits oviposition under laboratory and semi-field conditions. The manipulation of the odour-mediated oviposition site selection behaviour of gravid adults has distinct potential as a tool in the future control of malaria vectors. Here, we take a significant step toward the development of a long-sought-after control tool for gravid malaria mosquitoes, by identifying the first blend of odours to be used as a synthetic lure.

## Results

### Gravid malaria mosquitoes are attracted to rice odour

**Figure 1:**
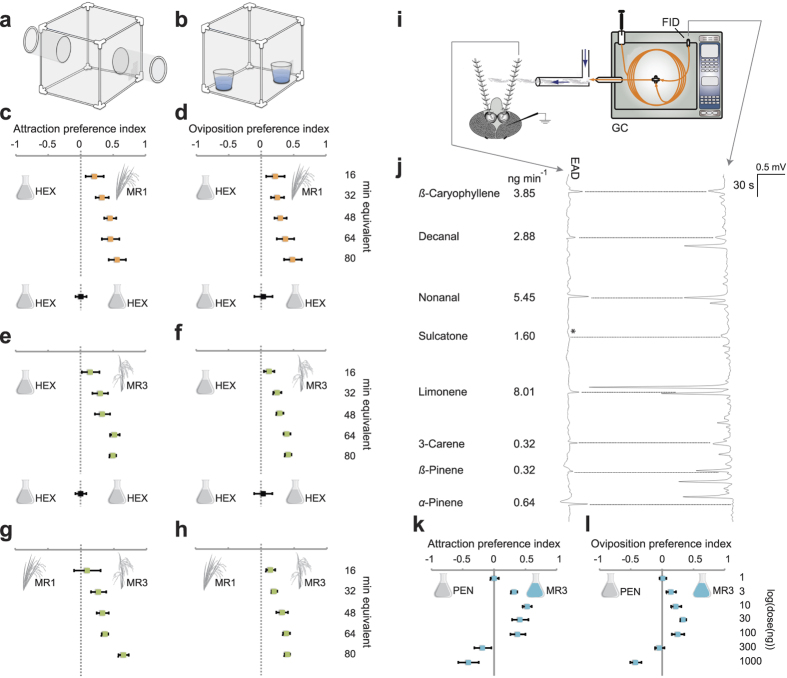
Gravid *Anopheles arabiensis* respond to rice odour. Diagrams of the two-port olfactometer (**a**) and oviposition (**b**) assays. Attraction (**c,e**) and oviposition (**d,f**) preference of mosquitoes to headspace volatiles of the MR1 and MR3 rice cultivars compared to hexane control, respectively (**c)***χ*^2^ = 11.87, P = 0.0006; (**d**), *χ*^2^ = 16.21, P < 0.0001; (**e**), *χ*^2^ = 8.378, P = 0.0038; (**f**), *χ*^2^ = 14.05, P = 0.0002). The headspace of the MR3 rice cultivar significantly attracted (**g**) and elicited oviposition (**h**) of mosquitoes over that of the MR1 cultivar (**g,** *χ*^2^ = 7.080, P = 0.0078; **h,** *χ*^2^ = 5.822, P = 0.0158). (**i**) Schematic of the combined gas chromatograph (GC) and electroantennographic detection (EAD) analysis. FID, flame ionization detector (courtesy of Dr Majid Ghaninia). (**j**) EAD traces depict voltage changes (mV) in response to the bioactive compounds in the headspace of the MR3 rice cultivar, eluting from the GC and registered by the FID. Note that the intensity of the response to sulcatone was variable and denoted here by an asterisk in the GC-EAD trace. The identity and release rate of the bioactive compounds are shown at the left. A synthetic blend composed of the bioactive compounds identified, in their natural ratio (**j**), elicited attraction (**k**) and stimulated oviposition (**l**) in gravid mosquitoes in a dose-dependent manner (**k,** *χ*^2^ = 18.34, P < 0.0001; **l,** *χ*^2^ = 32.93, P < 0.0001). Error bars represent standard errors of the mean. Statistical significance was tested using nominal logistic regression (likelihood ratio test). Ten replicates of 10 mosquitoes each were used in each behavioural experiment. All diagrammatic representations, other than in **i**, are courtesy of Pixabay, an open source image database.

Searching female mosquitoes make choices at increasingly refined scales, from habitat finding to resource acceptance^37^. Here, we investigate the nature and origin of the cues regulating the oviposition site selection in rice cultivations by *An. arabiensis*. To investigate whether *An. arabiensis* respond behaviourally to the odour compounds present in the air surrounding rice, we collected the headspace odour from two available rice cultivars, MR1 and MR3, at three different phenological stages: tillering, booting and flowering. Gravid mosquitoes showed a significant dose-dependent attraction to the headspace odour extracts of both MR1 and MR3 cultivars in a two-port olfactometer bioassay (Fig. 1a). This finding was independent of phenological state, when compared to the hexane controls (Supplementary Fig. 1; Supplementary Table 1) and the headspace odour extract of the breeding water collected from natural larval habitats of *An. arabiensis* (Supplementary Fig. 2; Supplementary Table 2), and indicates that rice volatiles can attract these mosquitoes over the short range. Similarly, gravid mosquitoes preferred to lay their eggs in water treated with the headspace odour extracts of all phenological stages of the MR1 and MR3 cultivars over controls (Supplementary Figs 3 and 4; Supplementary Tables 3 and 4) in a two-choice oviposition assay (Fig. 1b).

Since no significant differences were observed among the behavioural responses to the headspace of the phenological stages of either of the cultivars (Supplementary Figs 1–4; Supplementary Tables 1–4), the headspace extracts from all stages were pooled for each cultivar to use in subsequent behavioural assays. While the pooled headspace of each cultivar was dose-dependently attractive and elicited oviposition when compared to hexane (Fig. 1c–f; Supplementary Table 5) and breeding water controls (Supplementary Figs 5 and 6; Supplementary Tables 6 and 7), the pooled MR3 headspace extract was preferred by gravid mosquitoes over that of MR1 in both assays (Fig. 1g, and Supplementary Fig. 6c; Supplementary Tables 5 and 7). The behavioural observations showed that gravid mosquitoes are both attracted and oviposit in response to rice odour, and capable of discriminating between odour profiles emitted by different rice cultivars.

### A complex odour blend drives mosquito oviposition behaviour

As olfactory cues are detected mainly by the antenna in mosquitoes, we next tested the response of gravid mosquitoes to individual rice odour compounds of the preferred MR3 pooled headspace extract using combined gas chromatography and electroantennographic detection (GC-EAD; Fig. 1i). Of the eight compounds eliciting antennal responses (Fig. 1j), ß-caryophyllene, decanal, sulcatone (6-methyl-5-hepten-2-one) and limonene were previously identified in other rice cultivars^38^. The antennal response to sulcatone was variable across trials (* in Fig. 1j), but was included in further behavioural experiments to validate its role as an active component of the natural blend. The overall volatile release rate was 24 ng min^−1^, with limonene and nonanal as the most abundant compounds (Fig. 1j).

A synthetic blend of all the eight GC-EAD-active compounds identified in the MR3 headspace, in their natural ratio (Fig. 1j), elicited short-range attraction and oviposition in gravid *An. arabiensis* at the intermediate doses tested (Fig. 1k,l; Supplementary Table 5). In contrast, at the highest doses, the mosquitoes were either indifferent to, or avoided, the treatment. The release rates and ratio of individual compounds within the blend, in each assay, were found to be consistent with the synthetic blend, as determined by headspace odour analysis (Supplementary Fig. 7). Subtraction of individual components from the full blend, released at the lowest effective dose tested (10 ng), did not significantly reduce the attraction of gravid females in the two-port olfactometer against a pentane control, when compared to the attraction response to the full blend, with the exception of the removal of nonanal (Supplementary Fig. 8; Supplementary Table 8). However, nonanal by itself was unable to elicit attraction equivalent to that of the full blend (Supplementary Fig. 8). In contrast, the oviposition response of gravid females was significantly reduced in the two-choice oviposition assay with a pentane control, when compared to the oviposition response to the full blend, for all the subtractive blends, except ß-pinene (Supplementary Fig. 9; Supplementary Table 9). Thus, to induce the full behavioural repertoire, the complete blend is required.

### Gravid mosquitoes show long-range attraction to the rice blend

**Figure 2:**
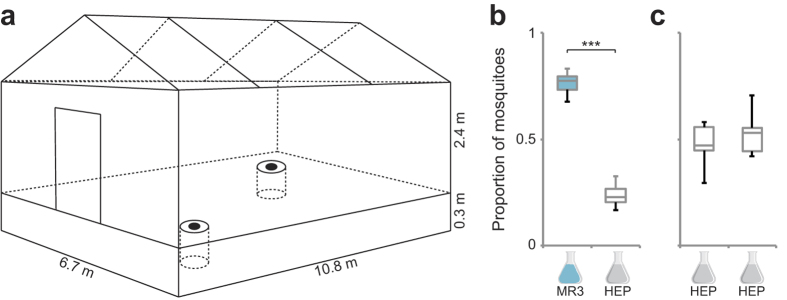
Long-range attraction of gravid *Anopheles arabiensis* to the synthetic rice blend. Diagram of the semi-field screened-in enclosure and the recessed BG-Sentinel traps (**a**) used to attract and capture free-flying mosquitoes. Using a randomised complete block design, a significantly higher number of mosquitoes were found to be attracted to traps baited with the synthetic blend compared to a heptane control (**b,** Pr = 0.74, 95% CI 0.68–0.79). No significant difference was observed between heptane controls (**c,** Pr = 0.51, 95% CI 0.44–0.58). Semi-field data (**b,c**) were analysed with generalized linear model using a quasi-binomial distribution. The experiments were conducted over 12 nights each with 200 gravid *An. arabiensis* released *per* trial.

An evaluation of the synthetic MR3 odour blend, in large (10.8 m × 6.7 m × 2.4 m) outdoor screened-in enclosures (Fig. 2a) in Kenya, revealed that oviposition site-seeking *An. arabiensis* were attracted over the long-range and caught in a dose-dependent manner in recessed BG sentinel traps^39^ (Supplementary Fig. 10). Based on this experiment, the most cost-effective release rate tested (3 ng min^−1^, based on the release rate of α-pinene) was used in further trials. Gravid *An. arabiensis* were significantly attracted to the full synthetic blend (Fig. 2b) when compared against the solvent control (heptane). No significant difference was found between the solvent controls when tested in a pairwise fashion (Fig. 2c).

## Discussion

Gravid *An. arabiensis* are attracted and stimulated to oviposit by rice plant volatiles under laboratory and semi-field conditions. This has the potential of strengthening the spatial association between the malaria vector and the agricultural landscape. Additional experiments are, however, required to verify that malaria mosquitoes use rice odours to locate appropriate oviposition sites in the wild. Female mosquitoes were attracted to volatiles emitted by all stages of the rice plant, which concurs with field observations showing that the larvae are present in rice cultivation throughout the growing season (s), with varying abundance^27^,^33^. Rice volatiles thus can play a significant role in the maintenance of vector populations, along with other aspects of rice cultivation that modulate female choice, including water availability, insecticide- and fertiliser-use, and vegetation density creating obstruction and shade^24^,^27^,^33^,^34^,^35^,^36^. The selective advantage for this association is yet to be conclusively determined. The enhanced availability of food in rice fields due to microbial growth in response to nitrogen surplus and water turbidity following fertiliser application^33^, along with the reduction of competition and predators, by insecticide treatment^34^, are possible drivers for this adaptive selection.

Mosquitoes use volatiles originating from plants as attractants and stimulants for oviposition^40^. While gravid anopheline mosquitoes display preferences for volatiles associated with living plants^41^ and microbes associated with their larval habitats^19^,^20^, culicines prefer those associated with the microbial fermentation of plant tissues^25^,^42^. Here, we have identified a long-range plant odour-based attractant blend of readily available and affordable organic compounds for gravid malaria mosquitoes, providing the integrated vector management community with new perspectives for developing a tool that targets gravid anophelines regardless of feeding and resting preferences. The revelation that rice plant volatiles attract gravid anophelines, together with the suitability of irrigated cereal cultivations as larval habitats^20^,^21^,^22^, strongly indicates that further analysis of the oviposition ecology of anophelines within these agro-ecological habitats is likely to produce additional targets for the development of odour-based attractants. While the identified blend consists of numerous ubiquitous plant compounds, previously shown to drive host plant selection in herbivorous^43^,^44^, the composition and ratio of this blend is novel, and it is this combination that results in the efficacy of this blend affecting malaria mosquito behaviour. The current malaria vector control strategies in widespread use, the IRS and LLINs, which focus on indoor feeding and resting mosquitoes, may benefit greatly from the addition of a complementary odour-based tool targeted against gravid females. As the next step following this pre-field study, we intend to test the rice odour blend under field conditions to evaluate its efficacy in relation to natural odours in the landscape.

## Materials and Methods

### Animal model

*Anopheles arabiensis*, Mwea and Dongola strains, were used for behavioural (International Centre of Insect Physiology and Ecology, Kenya; ICIPE) and electrophysiological (SLU, Sweden) analysis, respectively. The mosquitoes were maintained at 27 ± 2 °C, 75 ± 5% relative humidity under a 12 h light/12 h dark photoperiod. For laboratory experiments, larvae were reared in distilled water and fed Tetramin^®^ fish food (Tetra, Melle, Germany). Alternatively, for semi-field experiments, larvae were reared in water from Lake Victoria and fed on ground Go-Cat^®^ food (Nestlé Purina Petcare company, Nairobi, Kenya). Adults were maintained in cages (30 cm × 30 cm × 30 cm; custom made or Bugdorm, MegaView Science, Talchung, Taiwan), kept under laboratory (27 ± 2 °C, 75 ± 5% relative humidity) or ambient outdoor conditions (ICIPE, Mbita, Kenya), and provided with 10% honey or sucrose solution *ad libitum.* Five days post-emergence, females were offered sheep blood from an artificial feeder (SLU, Hemotek, Discovery Workshops, Accrington, UK), rat blood (ICIPE, Nairobi) or the arm of a volunteer (ICIPE, Mbita) once every day for two days, 15–30 min *per* day. For all experiments, gravid females, 3 days post-blood feeding, were selected by visually inspecting the enlarged pale white abdomen and used for bioassays.

### Headspace volatile collections

Headspace volatiles of MR1 and MR3 rice cultivars (150 replicates per stage) were collected in Saudi Star Agriculture and Irrigation Project in Gambella, Ethiopia. These available cultivars have desirable agronomic characters, including high yield, aeration seeding, moderate resistance to lodging, and are able to grow under rain-fed and irrigated conditions. The above-ground parts of intact rice plants in the field, at tillering, booting and flowering stages were enclosed in polyamide bags (Toppits, Cofresco, Germany). In addition, the headspace volatiles of water from natural breeding sites (200 replicates) were collected from 1 l poured into a Teflon bag. These sites were selected from pools at the edge of Lake Ziway, Ethiopia that contained *Anopheles gambiae sensu lato* larvae. A charcoal-filtered continuous airstream (1.0 l min^−1^) was drawn by a Personal Air Sampler (PAS-500, Spectrex, Redwood City, CA, USA) over the rice plant, or by a diaphragm vacuum pump (KNF Neuberger, Freiburg, Germany) over the breeding water, onto an aeration column for 2 h. Aeration columns were made of Teflon tubing (6 cm × 3 mm id), holding 35 mg Super Q (80/100 mesh; Alltech, Deerfield, IL, USA) between polypropylene wool plugs and Teflon stoppers. The columns were rinsed with 1 ml re-distilled *n*-hexane (LabScan, Malmö, Sweden) before use. Adsorbed volatiles were eluted with 300 μl re-distilled *n*-hexane, in order not to dilute the sample, or risk evaporation, between aeration and subsequent GC-EAD and GC-MS analyses. Headspace volatile extracts from each cultivar and phenological stage were pooled separately and then stored in glass vials at −80 °C until used for behavioural, electrophysiological and chemical analyses.

### Two-port olfactometer

A two-port still air olfactometer was used to test the mosquito attraction preference for the headspace volatiles collected from the different phenological stages of the MR1 and MR3 cultivars, and from natural breeding water. All assays were conducted between 18:00 and 21:00 local time under red light (200 lx) conditions (icipe, Nairobi), the peak oviposition activity as determined in pilot experiments. For each replicate, 10 gravid females were allowed to acclimatize for 5 min in a custom-made cage (22 cm × 30 cm × 12 cm; L:D:H) constructed of clear vinyl for easy viewing. Thereafter, two dental-wick odour dispensers (4 cm × 1 cm; L:d; DAB Dental AB, Upplands Väsby, Sweden) were simultaneously introduced into the cylindrical vinyl arms (13 cm × 9 cm; L:d) positioned at opposite ends of the cage. The ends of the cylindrical arms were covered by mesh. Mosquito attraction preference to the following treatments was analysed: (a) headspace volatiles of breeding water *vs.* hexane control, (b) headspace volatiles of each phenological stage of MR1 or MR3 *vs.* hexane, (c) headspace volatiles of each phenological stage of MR1 or MR3 *vs.* headspace volatiles of breeding water, and (d) headspace volatiles of the pooled phenological stages of MR1 *vs.* headspace volatiles of the pooled phenological stages of MR3. No significant difference in attraction preference was found between the headspace breeding water when compared directly against the hexane control. The behavioural response to the two rice cultivars was analysed to increasing release rates of the headspace volatile extract from all phenological stages of MR1 and MR3. After five minutes, the period after which there was no net increase in mosquitoes in each arm, the behavioural responses of the mosquitoes were scored by counting the number of mosquitoes in each port after five minute. Ten replicates *per* treatment and *per* dose were performed. Between each trial the bioassay was cleaned with 70% ethanol and the position of the treatments changed to avoid bias.

### Oviposition bioassay

The oviposition preference of gravid mosquitoes was analysed in a two-choice assay. Custom-made metal wire framed cages (30 cm × 30 cm × 30 cm) covered with white nylon mosquito netting were used. Two 100 ml polypropylene cups (Qingdao Ori-Color Industry and Commerce Co., Ltd., China), placed in opposite corners of the cages, and filled to the brim with distilled water or field collected breeding water, served as the oviposition substrate, as indicated. The position of the cups was exchanged between experiments. Treatment cups were conditioned by dispensing the headspace volatile extracts of the three phenological stages of MR1 and MR3 as described above directly onto the oviposition substrate; hexane was used as a control. An additional comparison between the headspace volatiles of the pooled phenological stages of MR1 *vs.* headspace volatiles of the pooled phenological stages of MR3 conditioned breeding water was performed for the oviposition assays. No significant difference in oviposition preference was found between the breeding and distilled water when compared directly against each other. Ten gravid mosquitoes were transferred from the maintenance cage at dusk (18:00), and the numbers of eggs in the two cups were counted on the following day (09:00). All experiments were replicated ten times.

### Electrophysiological analysis

Antennal responses of gravid female *An. arabiensis* to the pooled headspace extract of the three phenological stages of the MR3 rice cultivar were analysed using combined gas chromatography and electroantennographic detection (GC-EAD). An Agilent Technologies 6890 GC (Santa Clara, CA, USA) was equipped with a HP-5 column (30 m × 0.25 mm id, fused silica, 0.25 μm film thickness, Agilent Technologies), and hydrogen was used as the mobile phase at an average linear flow rate of 45 cm s^−1^. Each sample (2 μl) was injected in splitless mode (30 s, injector temperature 225 °C). The GC oven temperature was programmed from 35 °C (3 min hold) at 10 °C min^−1^ to 290 °C (10 min hold). At the GC effluent, 4 psi of nitrogen was added and split 1:1 in a Gerstel 3D/2 low dead volume four way-cross (Gerstel, Mülheim, Germany) between the flame ionization detector and the EAD. The GC effluent capillary for the EAD passed through a Gerstel ODP-2 transfer line, which tracked the GC oven temperature, into a glass tube (10 cm × 8 mm), where it was mixed with charcoal-filtered, humidified air (1.5 l min^−1^). The antenna was placed 0.5 cm from the outlet of this tube. The antennal preparation was made by using the excised head and inserting the distal end of the antenna, after cutting the distal segment, into a recording glass electrode filled with Beadle–Ephrussi Ringer. The recording electrode was connected to a pre-amplifier probe (10×) and then to a high impedance DC amplifier interface box (IDAC-2; Syntech, Kirchgarten, Germany). The reference electrode, filled with Beadle–Ephrussi Ringer, was inserted into the head capsule, and grounded. Fifteen recordings were performed.

### Chemical analysis

The pooled MR3 headspace extract was analysed on a combined gas chromatograph and mass spectrometer (GC-MS; 6890 GC and 5975 MS; Agilent Technologies), operated in the electron impact ionization mode at 70 eV. The GC was equipped with fused silica capillary columns (60 m × 0.25 mm, 0.25 μm film thickness), coated with DB-wax (J&W Scientific, Folsom, CA, USA) or HP-5MS (Agilent Technologies). Helium was used as the mobile phase at an average linear flow rate of 35 cm s^−1^. Two micro-litres of the sample were injected. The temperature programmes were the same as for the GC-EAD analysis. Compounds were identified according to retention times (Kovat’s indices) and mass spectra, in comparison with custom made and NIST05 libraries (Agilent), and confirmed by co-injection of authentic standards: (±)-*α*-pinene (CAS no. 7785-70-8; Aldrich, 98%), (−)-*β*-pinene (CAS no. 18172-67-3; Sigma, 99%), 3-carene (CAS no. 13466-78-9; Aldrich, 90%), (±)-limonene (CAS no. 5989-27-5; Sigma, 97%), nonanal (CAS no. 124-19-6; Aldrich, 95%), decanal (CAS no. 112-31-2; Aldrich, 92%), *β*-caryophyllene (CAS no. 87-44-5; Sigma, 98.5%) and sulcatone (6-methyl-5-hepten-2-one; CAS no. 110-93-0; Fluka, 96%). For quantification, 100 ng of heptyl acetate (99.8% chemical purity; Aldrich) was added as an internal standard to a 20 μl aliquot out of the total 400 μl headspace extract.

### Bioassays with synthetic blend

The assays were carried out in the same two-port olfactometer and oviposition bioassay that were used for the natural extract experiments (n = 10). The synthetic blend mimicked the composition and ratio of compounds in the pooled natural extracts of the MR3 rice cultivar (Fig. 1j). To verify the integrity of the blend, a 2 μl aliquot of the synthetic blend was dispensed as above. The head space was collected on an aeration column (as above) connected to a glass funnel (20 cm d), suspended over the cotton wick and oviposition substrate, through which air was drawn using a diaphragm vacuum pump (KNF Neuberger, Freiburg, Germany), at 50 ml min^−1^ for 30 min. The head space volatiles were analysed as above (Supplementary Fig. 7). Synthetic blends were prepared at different doses, in half orders of magnitude, between 1 to 1000 ng of α-pinene in pentane. The ratio among the compounds within the blend was maintained as a constant across all doses. Then, dose-response assays were conducted with the full blend against its solvent control. Subtractive bioassays followed in order to determine the relative activity of the identified components, after establishing the effective release rate (Fig. 1k,l).

### Semi-field trials with the synthetic blend

Semi-field experiments were conducted in an outdoor screened-in enclosure (10.8 m long × 6.7 m wide × 2.4 m high) at icipe, Thomas Odhiambo Campus, Mbita, Kenya. The ground was covered with sand to a depth of 30 cm for placement of modified BG Sentinel traps (Biogents AG, Regensburg, Germany)^45^. The traps, containing 4 l of distilled water, were recessed in the corners of the screened house at a distance of 1.5 m from the two adjacent walls. The experimental design used was a randomized complete block design and was performed under ambient environmental conditions. Preliminary trials were conducted to determine the attractiveness of the full synthetic blend dissolved in heptane (Merck, Darmstadt, DE), with release rates between 3 to 100 ng min^−1^ of α-pinene within the blend with four replications to identify the optimum release rate for the test system. The blend was released by diffusion from a wick dispenser made out of a 2 ml glass vial with a pin hole in the centre of the cap through which a cotton wick encased in Teflon protruded into the air^46^. The wick dispenser allows for the release of all compounds in constant proportions throughout the experiment. Heptane was used as a solvent to allow for the release of compounds throughout the course of the experiment. Two hundred gravid mosquitoes were released in the evening (18:00) and the number of females recaptured in each catch bag were counted at 08:00. Thereafter, trials were carried out using the most cost-effective release rate of 3 ng min^−1^ for 12 nights with the same number of gravid mosquitoes. Furthermore, controls vs. control trials were performed in which the attraction to both traps baited with heptane was evaluated, with the same number of mosquitoes and replications as above. Dissection of the females post-assay confirmed that 100% of the mosquitoes caught in the experiments were gravid.

### Statistical analysis

The attraction (AP) and oviposition (OP) preference for the laboratory assays were determined using indices generated by (T − C)/(T + C); whereas a proportion (Pr) was calculated for the semi-field bioassays: Pr = T(C)/(T + C). T is the number of mosquitoes or eggs associated with the test odours and C the number of mosquitoes or eggs associated with the control odours. The behavioural responses of gravid *An. arabiensis* in the two-port olfactometer and oviposition bioassay were analysed using a nominal logistic fit model, in which choice was the dependent variable, weighted by the number of (1) mosquitoes in the attraction assays and (2) eggs laid in the oviposition assays, with dose as the independent fixed effect and replicate as a random effect (JMP^®^ Pro 12.0.1. SAS Institute Inc., Cary, NC, USA). Here, we report the *χ*^2^ and *p*-value from the Likelihood Ratio Test. The data from semi-field experiments were analysed by using R software (version 3.00) with generalized linear model (GLZ) using a quasi-binomial distribution in R statistical software version 2.13^47^.

## Additional Information

**How to cite this article**: Wondwosen, B. *et al*. Rice volatiles lure gravid malaria mosquitoes, *Anopheles arabiensis.* *Sci. Rep.* **6**, 37930; doi: 10.1038/srep37930 (2016).

**Publisher's note:** Springer Nature remains neutral with regard to jurisdictional claims in published maps and institutional affiliations.

## Supplementary Material

Supplementary Information

## References

[b1] O’Meara W. P. . Changes in the burden of malaria in sub-Saharan Africa. Lancet Infect Dis 10, 545–555 (2010). 20637696 10.1016/S1473-3099(10)70096-7

[b2] Ranson H. & Lissenden N. Insecticide resistance in African *Anopheles* mosquitoes: A worsening situation that needs urgent action to maintain malaria control. Trends Parasitol 32, 187–196 (2016). 26826784 10.1016/j.pt.2015.11.010

[b3] Trape J.-F. . Malaria morbidity and pyrethroid resistance after the introduction of insecticide-treated bednets and artemisinin-based combination therapies: a longitudinal study. Lancet Infect Dis 11, 925–932 (2011). 21856232 10.1016/S1473-3099(11)70194-3

[b4] Ranson H. . Pyrethroid resistance in African anopheline mosquitoes: what are the implications for malaria control? Trends Parasitol 27, 91–98 (2011). 20843745 10.1016/j.pt.2010.08.004

[b5] Toé K. H. . Increased pyrethroid resistance in malaria vectors and decreased bed net effectiveness, Burkina Faso. Emerg Infect Dis 20, 1691–1696 (2014). 25279965 10.3201/eid2010.140619 PMC4193182

[b6] Zhou G. . Changing patterns of malaria epidemiology between 2002 and 2010 in Western Kenya: the fall and rise of malaria. PloS One 6, e20318 (2011). 21629783 10.1371/journal.pone.0020318 PMC3100336

[b7] Sinka M. E. . Modelling the relative abundance of the primary African vectors of malaria before and after the implementation of indoor, insecticide-based vector control. Malar J 15, 1 (2016). 26945997 10.1186/s12936-016-1187-8 PMC4779559

[b8] Riehle M. M. . A cryptic subgroup of *Anopheles gambiae* is highly susceptible to human malaria parasites. Science 331, 596–598 (2011). 21292978 10.1126/science.1196759 PMC3065189

[b9] Killeen G. F. . Eliminating malaria vectors. Parasit Vectors 6, 172 (2013). 23758937 10.1186/1756-3305-6-172 PMC3685528

[b10] Sougoufara S. . Biting by *Anopheles funestus* in broad daylight after use of long-lasting insecticidal nets: a new challenge to malaria elimination. Malar J 13, 10.1186 (2014). 24678587 10.1186/1475-2875-13-125 PMC3973838

[b11] Kitau J. . Species shifts in the *Anopheles gambiae* complex: do LLINs successfully control *Anopheles arabiensis*? PLoS One 7, e31481 (2012). 22438864 10.1371/journal.pone.0031481 PMC3306310

[b12] Russell T. L. . Linking individual phenotype to density-dependent population growth: the influence of body size on the population dynamics of malaria vectors. Proc R Soc Lond [Biol], rspb20110153 (2011). 10.1098/rspb.2011.0153 PMC3158942 21389034

[b13] Mwangangi J. M. . Shifts in malaria vector species composition and transmission dynamics along the Kenyan coast over the past 20 years. Malar J 12, 1–9 (2013). 23297732 10.1186/1475-2875-12-13 PMC3544599

[b14] Ojuka P. . Early biting and insecticide resistance in the malaria vector *Anopheles* might compromise the effectiveness of vector control intervention in Southwestern Uganda. Malar J 14, 1 (2015). 25879539 10.1186/s12936-015-0653-z PMC4416237

[b15] Fillinger U. & Lindsay S. W. Larval source management for malaria control in Africa: myths and reality. Malar J 10, 10.1186 (2011). 22166144 10.1186/1475-2875-10-353 PMC3273449

[b16] Okal M. N. . Water vapour is a pre-oviposition attractant for the malaria vector *Anopheles gambiae* sensu stricto. Malar J 12, 365 (2013). 24120083 10.1186/1475-2875-12-365 PMC3907035

[b17] Ogbunugafor C. B. & Sumba L. Behavioral evidence for the existence of a region-specific oviposition cue in *Anopheles gambiae* sensu stricto. J Vector Ecol 33, 321–324 (2008). 19263852 10.3376/1081-1710-33.2.321

[b18] Rejmánková E. . Volatile substances from larval habitats mediate species-specific oviposition in Anopheles mosquitoes. J Med Entomol 42, 95–103 (2005). 15799516 10.1093/jmedent/42.2.95

[b19] Sumba L. A. . Mediation of oviposition site selection in the African malaria mosquito *Anopheles gambiae* (Diptera: Culicidae) by semiochemicals of microbial origin. *Int J Trop* Insect Sci 24, 260–265 (2004).

[b20] Lindh J. M. . Discovery of an oviposition attractant for gravid malaria vectors of the *Anopheles gambiae* species complex. Malar J 14, 119 (2015). 25885703 10.1186/s12936-015-0636-0 PMC4404675

[b21] Ndenga B. A. . Productivity of malaria vectors from different habitat types in the western Kenya highlands. PLoS One 6, e19473 (2011). 21559301 10.1371/journal.pone.0019473 PMC3085476

[b22] Minakawa N. . Habitat characteristics of *Anopheles gambiae* sensu stricto larvae in a Kenyan highland. Med Vet Entomol 18, 301–305 (2004). 15347399 10.1111/j.0269-283X.2004.00503.x

[b23] FAO. Irrigation management transfer. FAO Water Reports 32 (2007).

[b24] Mwangangi J. M. . Survival of immature *Anopheles arabiensis* (Diptera: Culicidae) in aquatic habitats in Mwea rice irrigation scheme, central Kenya. Malar J 5, 114 (2006). 17125501 10.1186/1475-2875-5-114 PMC1698490

[b25] Ye-Ebiyo Y., Pollack R. J. & Spielman A. Enhanced development in nature of larval *Anopheles arabiensis* mosquitoes feeding on maize pollen. Am J Trop Med Hyg 63, 90–93 (2000). 11358003 10.4269/ajtmh.2000.63.90

[b26] Merritt R., Dadd R. & Walker E. Feeding behavior, natural food, and nutritional relationships of larval mosquitoes. Ann Rev Entomol 37, 349–374 (1992). 1347208 10.1146/annurev.en.37.010192.002025

[b27] Mwangangi J. M. . *Anopheles* larval abundance and diversity in three rice agro-village complexes Mwea irrigation scheme, central Kenya. Malar J 9, 10.1186 (2010). 20691120 10.1186/1475-2875-9-228 PMC2927610

[b28] Ijumba J. & Lindsay S. Impact of irrigation on malaria in Africa: paddies paradox. Med Vet Entomol 15, 1–11 (2001). 11297093 10.1046/j.1365-2915.2001.00279.x

[b29] Diakité N. R. . Spatial and temporal variation of malaria entomological parameters at the onset of a hydro-agricultural development in central Côte d’Ivoire. Malar J 14, 1–11 (2015). 26341670 10.1186/s12936-015-0871-4 PMC4560863

[b30] Mboera L. E. . Spatial abundance and human biting rate of *Anopheles arabiensis* and *Anopheles funestus* in savannah and rice agro-ecosystems of Central Tanzania. Geospat Health 10 (2015). 10.4081/gh.2015.322 26054517

[b31] Reid M. C. & McKenzie F. E. The contribution of agricultural insecticide use to increasing insecticide resistance in African malaria vectors. Malar J 15, 1 (2016). 26895980 10.1186/s12936-016-1162-4 PMC4759738

[b32] Darriet F., Rossignol M. & Chandre F. The combination of NPK fertilizer and deltamethrin insecticide favors the proliferation of pyrethroid-resistant *Anopheles gambiae* (Diptera: Culicidae). Parasite 19, 159–164 (2012). 22550627 10.1051/parasite/2012192159 PMC3671440

[b33] Mutero C. M. . Ammonium sulphate fertiliser increases larval populations of *Anopheles arabiensis* and culicine mosquitoes in rice fields. Acta Trop 89, 187–192 (2004). 14732240 10.1016/j.actatropica.2003.08.006

[b34] Service M. Mortalities of the immature stages of species B of the *Anopheles gambiae* complex in Kenya: comparison between rice fields and temporary pools, identification of predators, and effects of insecticidal spraying. J Med Entomol 13, 535–545 (1977). 845895 10.1093/jmedent/13.4-5.535

[b35] Jarju L. . Agriculture and the promotion of insect pests: rice cultivation in river floodplains and malaria vectors in The Gambia. Malar J 8, 170 (2009). 19635125 10.1186/1475-2875-8-170 PMC2734858

[b36] Muturi E. J. . Mosquito species diversity and abundance in relation to land use in a riceland agroecosystem in Mwea, Kenya. J Vector Ecol 31, 129–137 (2006). 16859101 10.3376/1081-1710(2006)31[129:msdaai]2.0.co;2

[b37] Takken W. & Knols B. G. Odor-mediated behavior of Afrotropical malaria mosquitoes. Ann Rev Entomol 44, 131–157 (1999). 9990718 10.1146/annurev.ento.44.1.131

[b38] Fujii T., Hori M. & Matsuda K. Attractants for rice leaf bug, *Trigonotylus caelestialium* (Kirkaldy), are emitted from flowering rice panicles. J Chem Ecol 36, 999–1005 (2010). 20680414 10.1007/s10886-010-9839-6

[b39] Okal M. N. . Analyzing chemical attraction of gravid *Anopheles gambiae* sensu stricto with modified BG-Sentinel traps. Parasit Vectors 8, 301 (2015). 26036270 10.1186/s13071-015-0916-0 PMC4456765

[b40] Afify A. & Galizia C. G. Chemosensory cues for mosquito oviposition site selection. J Med Entomol 52, 120–130 (2015). 26336295 10.1093/jme/tju024

[b41] Torres-Estrada J. L. . Vegetation-derived cues for the selection of oviposition substrates by *Anopheles albimanus* under laboratory conditions. J Am Mosq Control Assoc 21, 344–349 (2005). 16506557 10.2987/8756-971X(2006)21[344:VCFTSO]2.0.CO;2

[b42] Herrera-Varela M. . Habitat discrimination by gravid *Anopheles gambiae* sensu lato–a push-pull system. Malar J 13, 1 (2014). 24693951 10.1186/1475-2875-13-133 PMC3975139

[b43] Knudsen J. T., Tollsten L. & Bergström L. G. Floral scents – a checklist of volatile compounds isolated by head-space techniques. Phytochemistry 33, 253–280 (1993).

[b44] Dudareva N. . Plant volatiles: recent advances and future perspectives. Crit Rev Plant Sci 25, 417–440 (2006).

[b45] Okal M. N. . Analysing the oviposition behaviour of malaria mosquitoes: design considerations for improving two-choice egg count experiments. Malar J 14, 1–17 (2015). 26088669 10.1186/s12936-015-0768-2 PMC4474426

[b46] Birgersson G. . Pheromone production, attraction, and interspecific inhibition among four species of Ips bark beetles in the Southeastern USA. Psyche 2012 (2012).

[b47] Team R. C. R.: a language and environment for statistical computing. Vienna, Austria: R Foundation for Statistical Computing. URL http://www.R-project.org (2013).

