# Supplementary material for: Rice volatiles lure gravid malaria mosquitoes, Anopheles arabiensis
Source: Sci Rep. 2016 Nov 30;6:37930. doi: 10.1038/srep37930 (PMC5128813; doi:10.1038/srep37930)
Supplement: Supplementary Information [file srep37930-s1.pdf]

Rice volatiles lure gravid malaria mosquitoes, *Anopheles arabiensis*

Betelehem Wondwosen[1,2,3], Göran Birgersson[2], Emiru Seyoum[1], Habte Tekie[1], Baldwyn Torto[3], Ulrike Fillinger[3,4], Sharon R Hill[2§], and Rickard Ignell[2§*]

[1]Department of Zoological Sciences, Addis Ababa University, P. O. Box 1176, Addis Ababa, Ethiopia.

[2]Unit of Chemical Ecology, Department of Plant Protection Biology, Swedish University of Agricultural Sciences, P. O. Box 102, Sundsvägen 14, 230 53 Alnarp, Sweden.

[3]Behavioural and Chemical Ecology Department, International Centre of Insect Physiology and Ecology, P. O. Box 30772, Nairobi 00100, Kenya

[4]Disease Control Department, London School of Hygiene and Tropical Medicine, London WC1E 7HT, UK

[§]Shared authorship

[*]Corresponding author, rickard.ignell@slu.se

Supplementary Figure Legends

Supplementary Figure 1. **Attraction preference of *Anopheles arabiensis* to the booting, tillering and flowering stages of the MR1 (a, c, e) and MR3 (b, d, f) rice cultivars against hexane controls (HEX).** Headspace volatiles of the three phenological stages of MR1 and MR3 rice cultivars elicited release rate-dependent attraction (**a**, χ^2=6.104, P=0.0135; **b**, χ^2=13.55, P=0.0002; **c**, χ^2=14.02, P=0.0002; **d**, χ^2=13.13, P=0.0003; **e**, χ^2=8.370, P=0.0038; **f**, χ^2=8.349, P=0.0039). No significant difference in attraction was found between the phenological stages of either cultivar (MR1, Release rate, χ^2=32.74, P<0.0001, Stage, χ^2=0.2267, P=0.8928; MR3, Release rate, χ^2=29.53, P<0.0001, Stage, χ^2=0.3283, P=0.8486). Statistical significance was tested using nominal logistic regression (likelihood ratio test). Ten replicates, of 10 gravid mosquitoes each, were used for every behavioural experiment. Error bars represent standard errors of the mean.

Supplementary Figure 2. **Attraction preference of *Anopheles arabiensis* to the booting, tillering and flowering stages of the MR1 (a, c, e) and MR3 (b, d, f) rice cultivars against the headspace of breeding water (BW).** Headspace volatiles of the three phenological stages of MR1 and MR3 rice cultivars elicited release rate-dependent attraction (**a**, χ^2=9.628, P=0.0019; **b**, χ^2=13.18, P=0.0003;

c, χ^2=8.207, P=0.0042; **d**, χ^2=21.30, P<0.0001; **e**, χ^2=18.26, P<0.0001; **f**, χ^2=6.083, P=0.0136). No significant difference in attraction was found between the phenological stages of either cultivar (MR1, Release rate, χ^2=30.63, P<0.0001, Stages, χ^2=0.3287, P=0.8485; MR3, Release rate, χ^2=42.90, P<0.0001, Stages, χ^2=0.2532, P=0.8811). Statistical significance was tested using nominal logistic regression (likelihood ratio test). Ten replicates, of 10 gravid mosquitoes each, were used for every behavioural experiment. Error bars represent standard errors of the mean.

Supplementary Figure 3. **Oviposition preference of *Anopheles arabiensis* to the booting (a, b), tillering (c, d) and flowering (e, f) stages of the MR1 (left) and MR3 (right) rice cultivars against hexane controls (HEX).** Headspace volatiles of the three phenological stages of MR1 and MR3 rice cultivars added to distilled water elicited release rate-dependent oviposition (**a,** χ^2=14.58, P<0.0001; **b,** χ^2 =10.02, P=0.0015; **c,** χ^2=9.607, P=0.0019; **d,** χ^2=9.959, P=0.0016; **e,** χ^2=13.34, P=0.0003; **f,** χ^2=20.57, P<0.0001). No significant difference in oviposition was found between the phenological stages of either cultivar (MR1**,** Release rate, χ^2=33.90, P<0.0001, Stage, χ^2=0.5059, P=0.7765; MR3, Release rate, χ^2=42.89, P<0.0001, Stage, χ^2=5.426, P=0.0663). Statistical significance was tested using nominal logistic regression (likelihood ratio test). Ten replicates, of 10 gravid mosquitoes each, were used for every behavioural experiment. Error bars represent standard errors of the mean.

Supplementary Figure 4. **Oviposition preference of *Anopheles arabiensis* to the booting (a, b), tillering (c, d) and flowering (e, f) stages of the MR1 (left) and MR3 (right) rice cultivars against hexane controls in breeding water (BW).** Headspace volatiles of the three phenological stages of MR1 and MR3 rice cultivars added to breeding water elicited release rate-dependent oviposition (**a,** χ^2=10.18, P=0.0014; **b,** χ^2=9.631, P=0.0019; **c,** χ^2=9.837, P=0.0017; **d,** χ^2=9.200, P=0.0024; **e,** χ^2=6.262, P=0.0123; **f,** χ^2=10.03, P<0.0015). No significant difference in oviposition was found between the phenological stages of either cultivar (MR1, Release rate, χ^2=29.64, P<0.0001, Stage, χ^2=2.563, P=0.2776; MR3, Release rate, χ^2=25.19, P<0.0001, Stage, χ^2=5.686, P=0.0582). Statistical significance was tested using nominal logistic regression (likelihood ratio test). Ten replicates, of 10 gravid mosquitoes each, were used for every behavioural experiment. Error bars represent standard errors of the mean.

Supplementary Figure 5. **Attraction preference of *Anopheles arabiensis* to the pooled headspace of MR1 (a) and MR3 (b) rice cultivars compared to the headspace of breeding water (BW).** Headspace volatiles of the MR1 and MR3 rice cultivars elicited a release rate-dependent attraction to the headspace of the breeding water control (**a,** χ^2=11.51, P=0.0007; **b,** χ^2=11.88, P=0.0006). Statistical significance was tested using nominal logistic regression (likelihood ratio test). Ten replicates, of 10 gravid mosquitoes each, were used for every behavioural experiment. Error bars represent standard errors of the mean.

Supplementary Figure 6. **Oviposition preference of *Anopheles arabiensis* to the pooled headspace of MR1 (a) and MR3 (b) rice cultivars compared to hexane in breeding water (BW) and between the headspace of the cultivars in BW (c).** Headspace volatiles of the MR1 and MR3 rice cultivars elicited a release rate-dependent oviposition to the headspace of the breeding water control (**a,** χ^2=11.34, P=0.0008; **b,** χ^2=7.538, P=0.0060; **c**, χ^2=4.740, P=0.0295). Statistical significance was tested using nominal logistic regression (likelihood ratio test). Ten replicates, of 10 gravid mosquitoes each, were used for every behavioural experiment. Error bars represent standard errors of the mean.

Supplementary Figure 7. **Relative release rates of individual components in the synthetic MR3 rice odour blend in the different assays.** Release rates were calculated as percentage of the release rate of α-pinene, after dispensing the full synthetic blend on water (green; oviposition assay) and by cotton wick (blue; attraction assay) and assaying the headspace; then comparing back to the full blend as synthesized (red).

Supplementary Figure 8. **Attraction preference of *Anopheles arabiensis* to the full and subtractive synthetic MR3 blends against pentane controls.** Overall, the subtractive blends were found to elicit an attraction comparable with the full blend (χ^2=14.68, P=0.1002). Statistical significance was tested using nominal logistic regression (likelihood ratio test) between each treatment and the full blend (*** P<0.001; ** P<0.01; * P<0.05). The attraction to the nonanal-reduced blend was significantly different from that to the full blend (Odds ratio=3.105, P=0.0022). Nonanal by itself was unable to elicit attraction equivalent to that of the full blend (Odds ratio=2.306, P=0.0225). Ten replicates, of

10 gravid mosquitoes each, were used for every behavioural experiment. Error bars represent standard errors of the mean. Box plots are shown with whiskers determined as Tukey inner fences.

Supplementary Figure 9. **Oviposition preference of *Anopheles arabiensis* to the full and subtractive synthetic MR3 blends against pentane controls in distilled water.** Overall, the subtractive blends elicited a positive oviposition preference compared with pentane (χ^2=22.09, P<0.0001), however, not to the level of the full blend (χ^2=4.82, P=0.0282). The oviposition response to the nonanal-reduced blend was significantly different from that to the full blend (Odds ratio=0.6510, P=0.0017). Statistical significance was tested using nominal logistic regression (likelihood ratio test) between each treatment and the full blend (*** P<0.001; ** P<0.01; * P<0.05). Ten replicates, of 10 gravid mosquitoes each, were used for every behavioural experiment. Box plots are shown with whiskers determined as Tukey inner fences.

Supplementary Figure 10. **Experiment to determine the effective release rate of the synthetic MR3 odour blend under semi-field conditions.** The release rates indicated are based on those of α-pinene in heptane. The ratio among the compounds within the blend was maintained as a constant across all doses. Statistical significance was tested with nominal logistic regression (likelihood ratio test) (χ^2=137.1, P<0.0001). Different lowercase letters indicate significant differences by Odds ratio pairwise comparisons (likelihood ratio test). Error bars denote standard error of the mean.

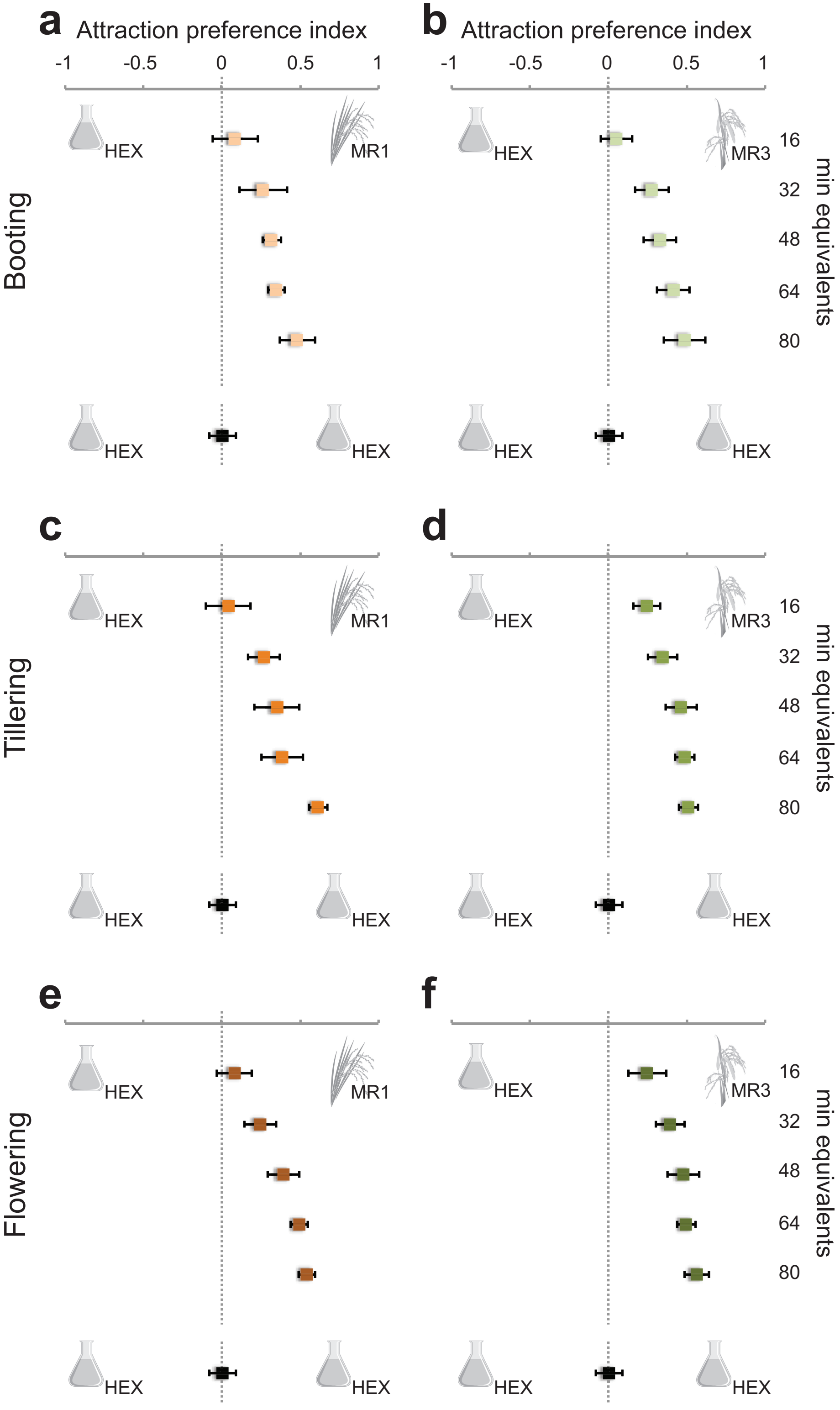

a
b
c
d
e
f
Attraction preference index
-1
-0.5
0
0.5
1
HEX
MR1
MR3
16
32
48
64
80
min equivalents
Booting
Tillering
Flowering

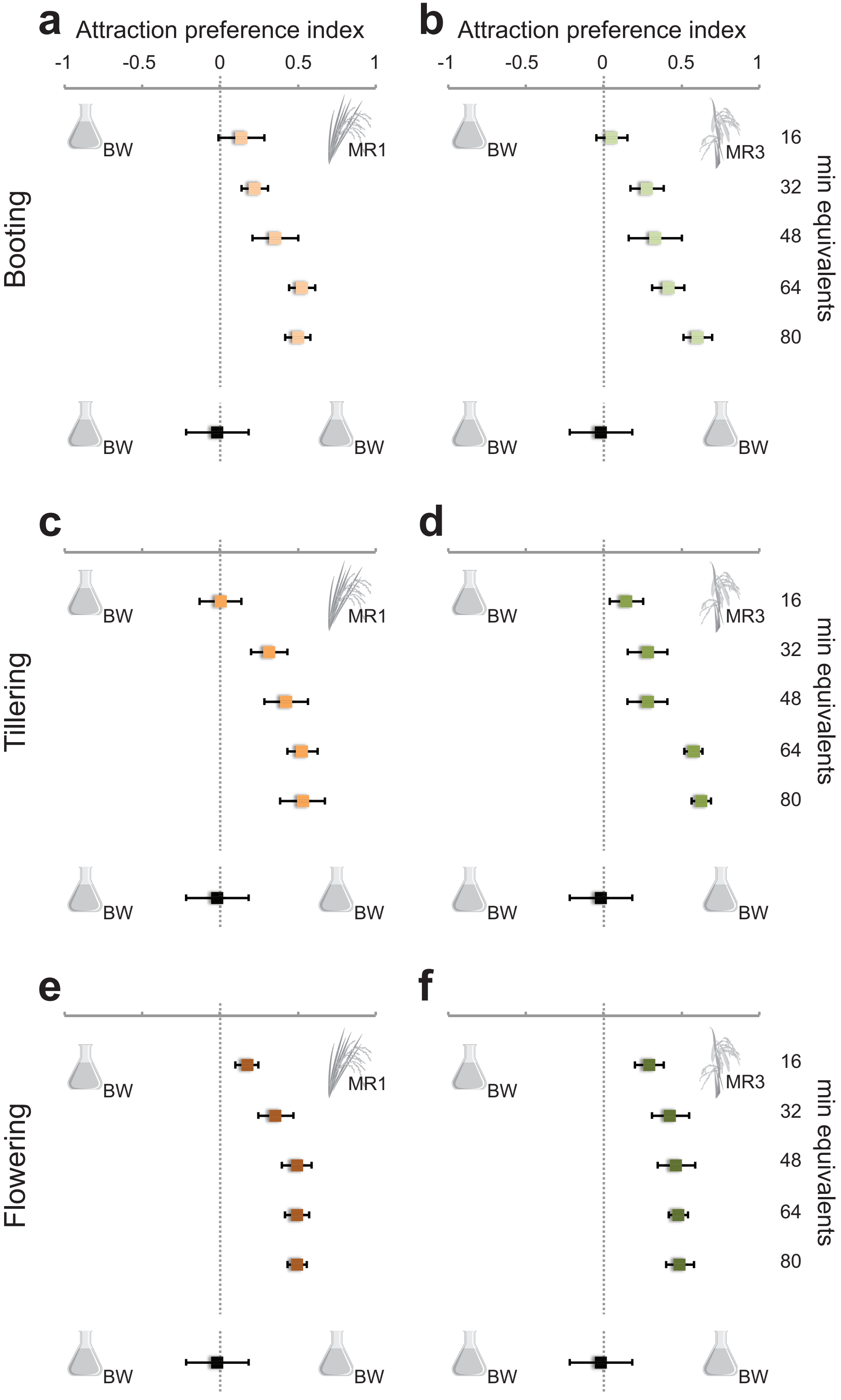
a
b
c
d
e
f
Attraction preference index
Attraction preference index
-1
-0.5
0
0.5
1
BW
MR1
MR3
min equivalents
16
32
48
64
80
Booting
Tillering
Flowering

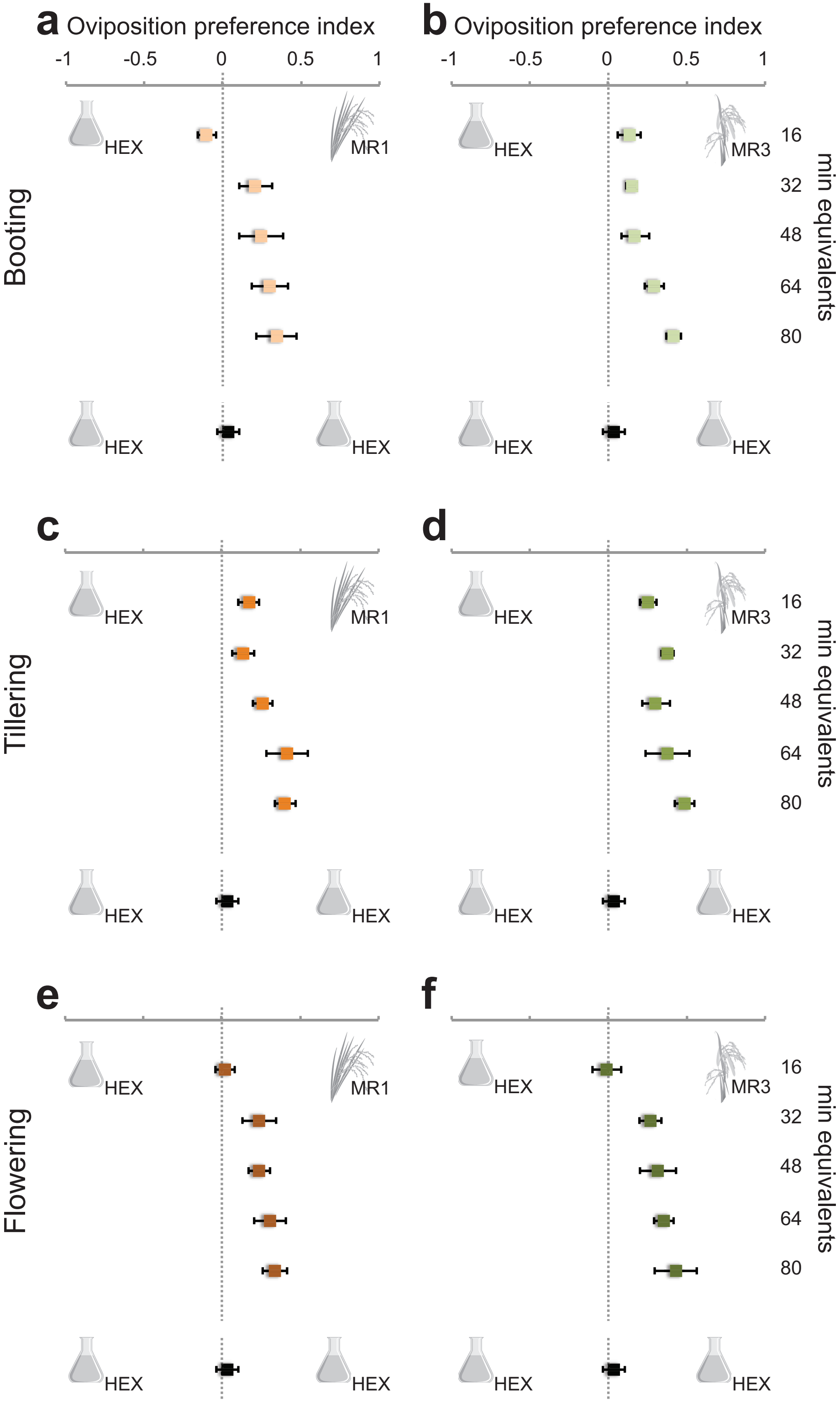
a Oviposition preference index
b Oviposition preference index
-1
-0.5
0
0.5
1
HEX
MR1
MR3
16
32
48
64
80
min equivalents
Booting
c
d
Tillering
e
f
Flowering

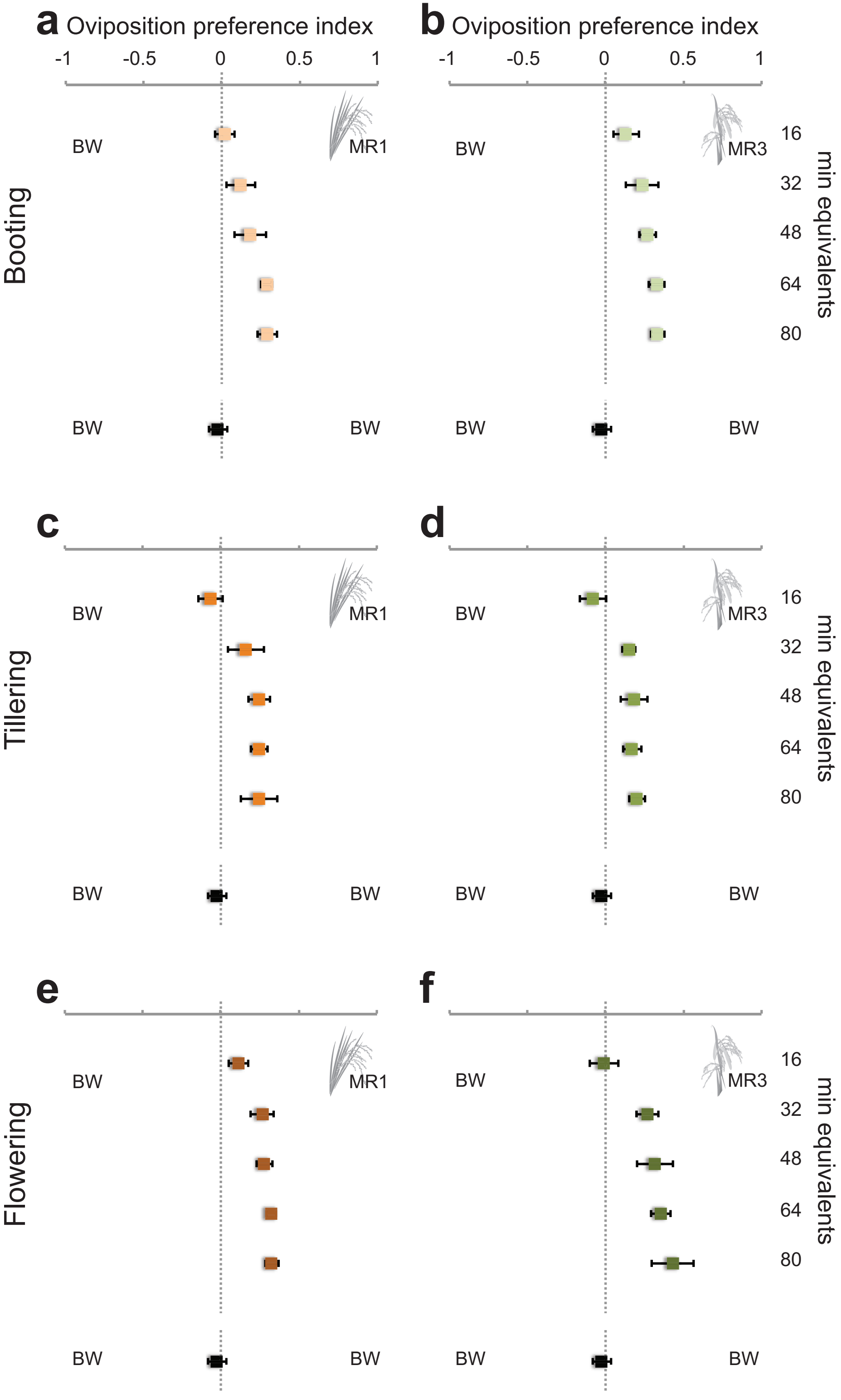

a
Oviposition preference index
b
Oviposition preference index
-1
-0.5
0
0.5
1
BW
MR1
BW
MR3
16
32
48
64
80
min equivalents
Booting
Tillering
Flowering
c
d
e
f

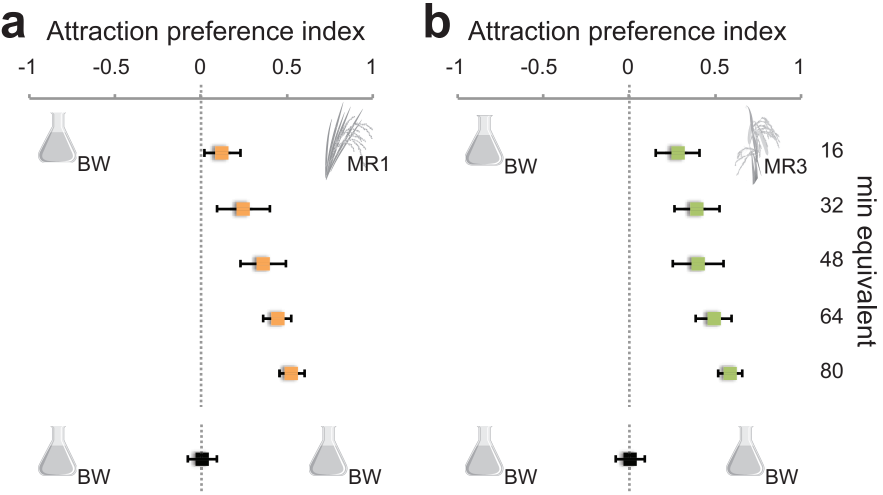
a
Attraction preference index
-1
-0.5
0
0.5
1
BW
MR1
BW
BW
b
Attraction preference index
-1
-0.5
0
0.5
1
BW
MR3
16
32
48
64
80
min equivalent
BW
BW

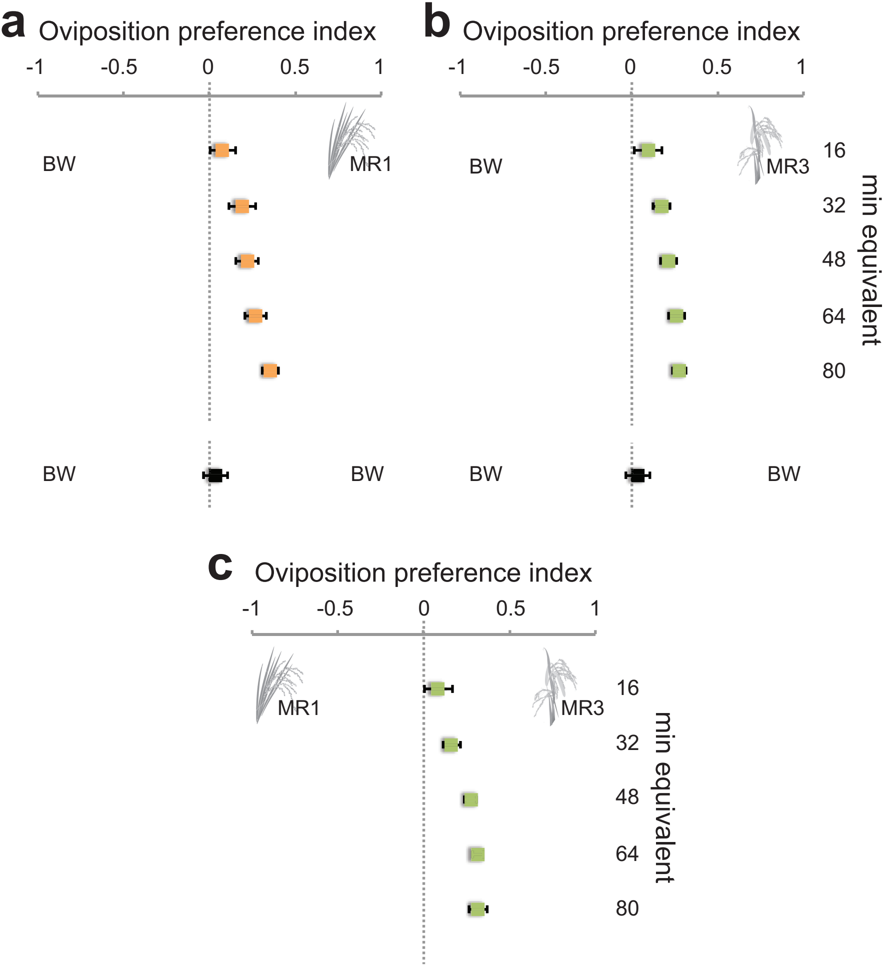

a
Oviposition preference index
-1
-0.5
0
0.5
1
BW
MR1
BW
BW
b
Oviposition preference index
BW
MR3
BW
BW
16
32
48
64
80
min equivalent
c
Oviposition preference index
MR1
MR3

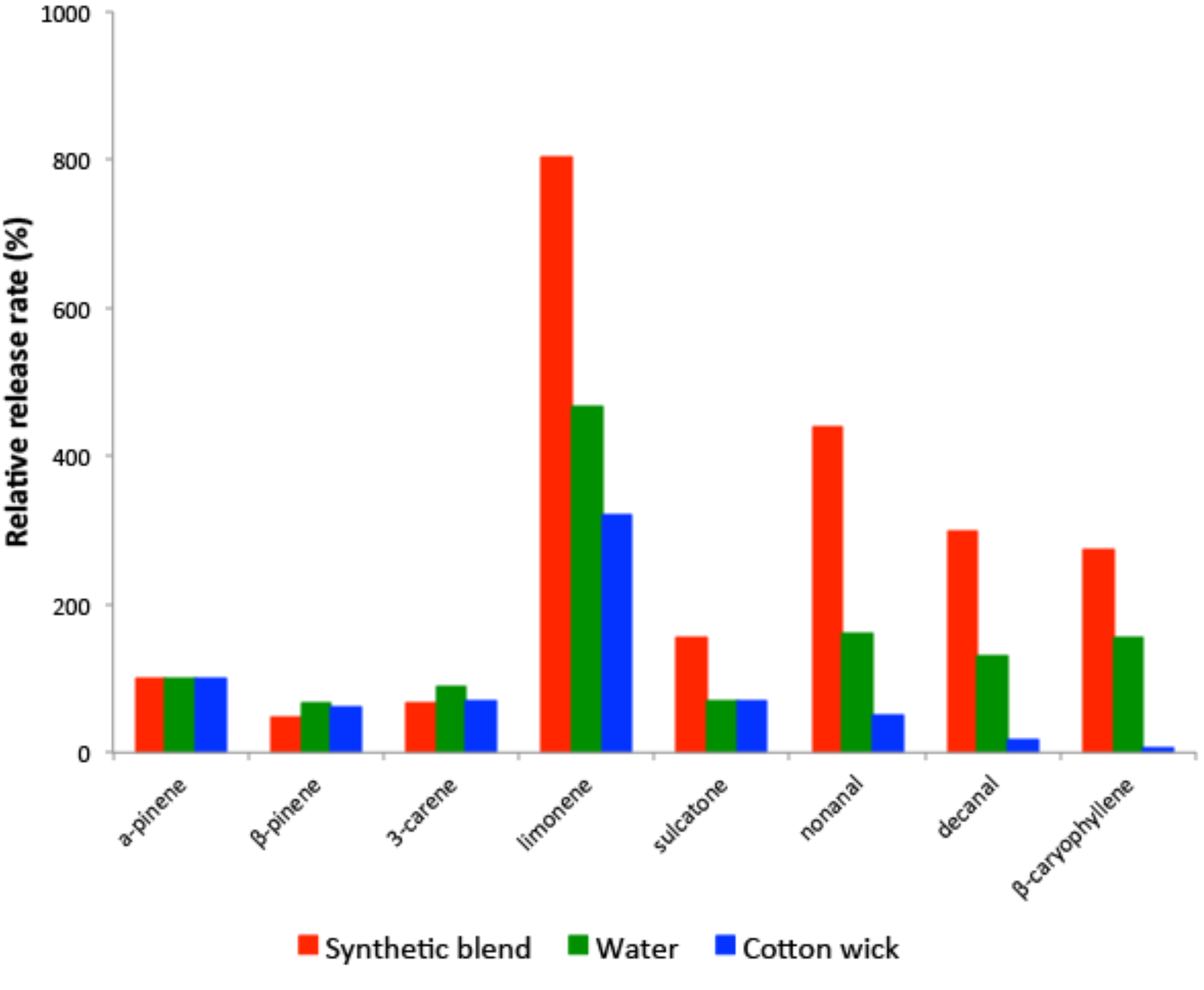

Relative release rate (%)
1000
800
600
400
200
0
a-pinene
β-pinene
3-carene
limonene
sulcatone
nonanal
decanal
β-caryophyllene
Synthetic blend
Water
Cotton wick

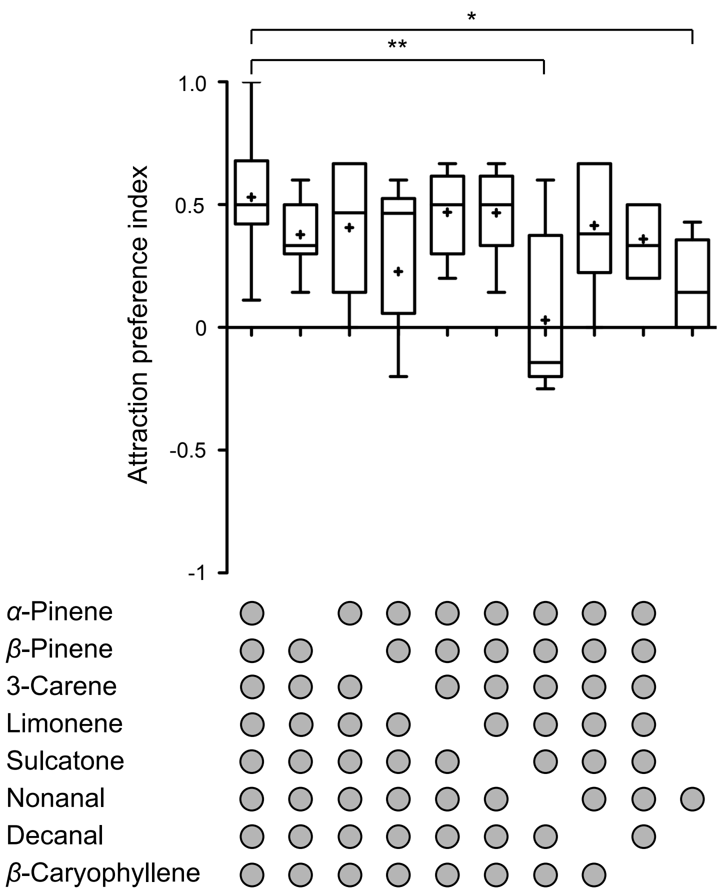

*
**
1.0
0.5
0
-0.5
-1
Attraction preference index
α-Pinene
β-Pinene
3-Carene
Limonene
Sulcatone
Nonanal
Decanal
β-Caryophyllene

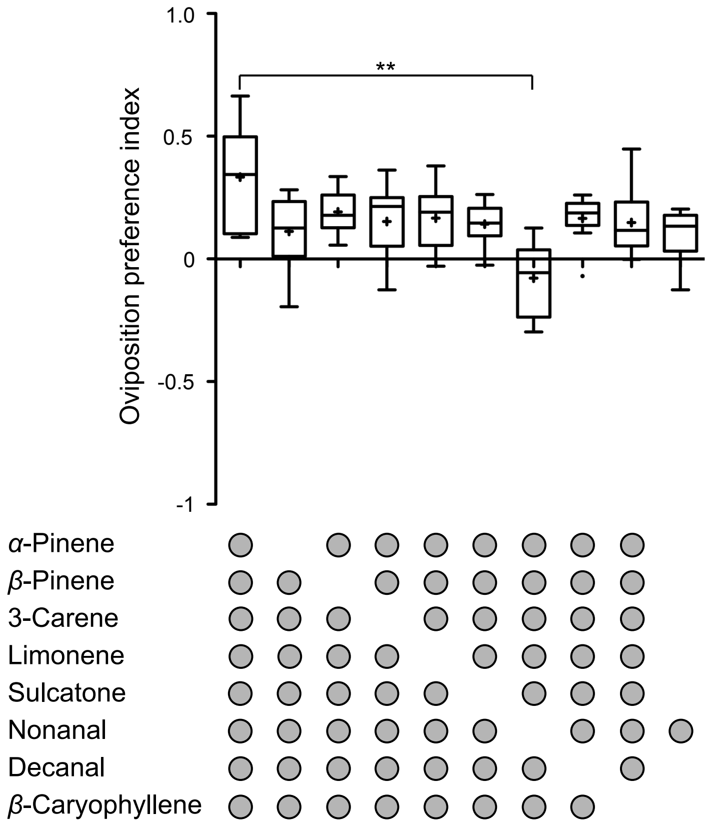
1.0
0.5
0
-0.5
-1
Oviposition preference index
**
α-Pinene
β-Pinene
3-Carene
Limonene
Sulcatone
Nonanal
Decanal
β-Caryophyllene

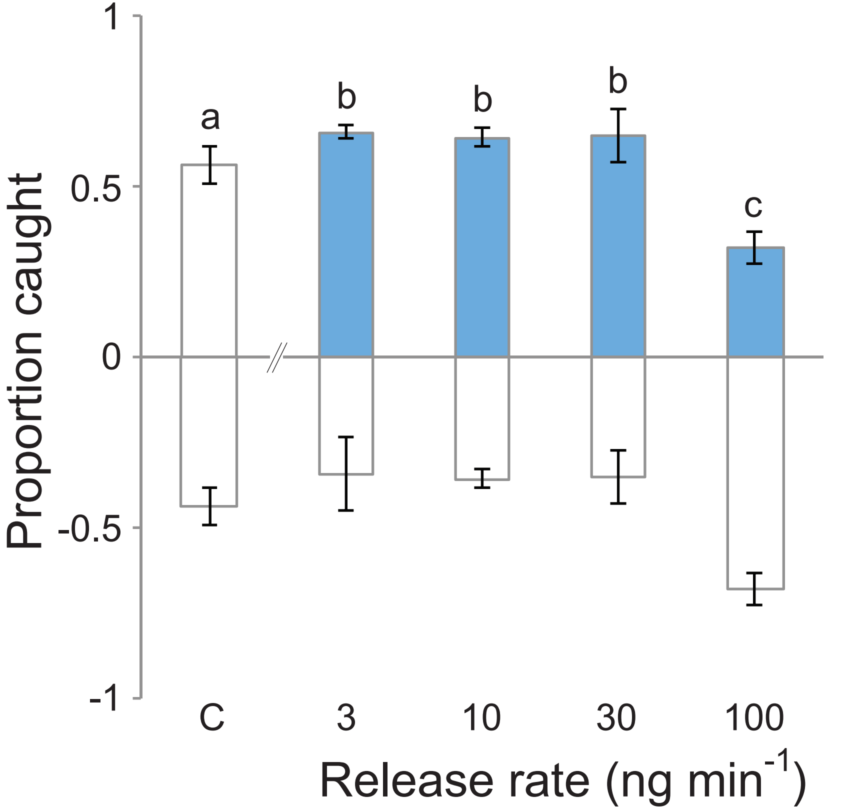

Proportion caught
1
0.5
0
-0.5
-1
a
b
b
b
c
C
3
10
30
100
Release rate (ng min-1)

Supplementary Table 1: Number of gravid *Anopheles arabiensis* responding in the attraction assay to extracts of rice cultivars at different phenological stages.

	Attraction response		
**Figure**	**Dose**	**Control**	**Test**
Suppl. Fig. 1a	*min equivalents*	*Hexane*	*Booting rice extract (MR1)*
	0	15	15
	16	18	26
	32	19	39
	48	21	42
	64	24	50
	80	16	50
Suppl. Fig. 1b	*min equivalents*	*Hexane*	*Booting rice extract (MR3)*
	0	15	15
	16	19	21
	32	25	42
	48	22	50
	64	16	48
	80	20	65
Suppl. Fig. 1c	*min equivalents*	*Hexane*	*Tillering rice extract (MR1)*
	0	15	15
	16	23	28
	32	25	40
	48	17	34
	64	21	46
	80	14	59
Suppl. Fig. 1d	*min equivalents*	*Hexane*	*Tillering rice extract (MR3)*
	0	15	15
	16	20	32
	32	22	39
	48	17	44
	64	18	50
	80	20	61

	Attraction response		
**Figure**	**Dose**	**Control**	**Test**
Suppl. Fig. 1e	*min equivalents*	*Hexane*	*Flowering rice extract (MR1)*
	0	15	15
	16	22	26
	32	21	34
	48	18	47
	64	18	53
	80	17	59
Suppl. Fig. 1f	*min equivalents*	*Hexane*	*Flowering rice extract (MR3)*
	0	15	15
	16	19	28
	32	19	39
	48	19	50
	64	18	52
	80	16	49

Supplementary Table 2: Number of gravid *Anopheles arabiensis* responding in the attraction assay to extracts of rice cultivars at different phenological stages.

	Attraction response		
**Figure**	**Dose**	**Control**	**Test**
Suppl. Fig. 2a	*min equivalents*	*Extract of breeding water*	*Booting rice extract (MR1)*
	0	13	11
	16	17	26
	32	23	37
	48	24	51
	64	18	51
	80	20	61
Suppl. Fig. 2b	*min equivalents*	*Extract of breeding water*	*Booting rice extract (MR3)*
	0	13	11
	16	19	21
	32	25	42
	48	22	50
	64	16	48
	80	17	70
Suppl. Fig. 2c	*min equivalents*	*Extract of breeding water*	*Tillering rice extract (MR1)*
	0	13	11
	16	19	21
	32	25	42
	48	22	50
	64	16	48
	80	17	48
Suppl. Fig. 2d	*min equivalents*	*Extract of breeding water*	*Tillering rice extract (MR3)*
	0	13	11
	16	21	28
	32	22	36
	48	21	44
	64	14	49
	80	13	68

	Attraction response		
**Figure**	**Dose**	**Control**	**Test**
Suppl. Fig. 2e		*Extract of breeding water*	*Flowering rice extract (MR1)*
	0	13	11
	16	23	34
	32	17	38
	48	18	48
	64	18	50
	80	21	62
Suppl. Fig. 2f	*min equivalents*	*Extract of breeding water*	*Flowering rice extract (MR3)*
	0	13	11
	16	20	31
	32	18	47
	48	21	53
	64	19	53
	80	20	56

Supplementary Table 3: Number of eggs laid in the oviposition assay by gravid *Anopheles arabiensis* to extracts of rice cultivars at different phenological stages.

	Oviposition response		
**Figure**	**Dose**	**Control**	**Test**
Suppl. Fig. 3a	*min equivalents*	*Hexane*	*Booting rice extract (MR1)*
	0	3738	3811
	16	5339	4396
	32	3624	5514
	48	3396	5920
	64	3506	6456
	80	3273	6595
Suppl. Fig. 3b	*min equivalents*	*Hexane*	*Booting rice extract (MR3)*
	0	3738	3811
	16	4092	5419
	32	4570	6129
	48	4290	5837
	64	3638	6570
	80	2816	6691
Suppl. Fig. 3c	*min equivalents*	*Hexane*	*Tillering rice extract (MR1)*
	0	3738	3811
	16	4076	5668
	32	4130	5581
	48	3647	6172
	64	3365	5780
	80	3037	6855
Suppl. Fig. 3d	*min equivalents*	*Hexane*	*Tillering rice extract (MR3)*
	0	3738	3811
	16	3675	6106
	32	3079	6722
	48	3271	5888
	64	2831	6129
	80	2404	7048

	Oviposition response		
Figure	Dose	Control	Test
Suppl. Fig. 3e	*min equivalents*	*Hexane*	*Flowering rice extract (MR1)*
	0	3738	3811
	16	4900	5062
	32	3883	5711
	48	3955	6304
	64	3486	6379
	80	3270	6201
Suppl. Fig. 3f	*min equivalents*	*Hexane*	*Flowering rice extract (MR3)*
	0	3738	3811
	16	5200	5111
	32	3777	6391
	48	3247	6298
	64	3340	6749
	80	2888	7192

Supplementary Table 4: Number of eggs laid in the oviposition assay by gravid *Anopheles arabiensis* to extracts of rice cultivars at different phenological stages.

	Oviposition response		
**Figure**	**Dose**	**Control**	**Test**
Suppl. Fig. 4a	*min equivalents*	*Extract of breeding water*	*Booting rice extract (MR1)*
	0	4902	4669
	16	4962	5137
	32	4378	5475
	48	4142	5922
	64	3613	6497
	80	3658	6633
Suppl. Fig. 4b	*min equivalents*	*Extract of breeding water*	*Booting rice extract (MR3)*
	0	4902	4669
	16	4315	5626
	32	3986	6201
	48	3795	6466
	64	3378	6501
	80	3294	6344
Suppl. Fig. 4c	*min equivalents*	*Extract of breeding water*	*Tillering rice extract (MR1)*
	0	4902	4669
	16	5350	4672
	32	4377	6002
	48	3896	6353
	64	3895	6333
	80	3824	6450
Suppl. Fig. 4d	*min equivalents*	*Extract of breeding water*	*Tillering rice extract (MR3)*
	0	4902	4669
	16	5932	5051
	32	4566	6198
	48	4240	6056
	64	4541	6489
	80	4331	6451

Oviposition response			
**Figure**	**Dose**	**Control**	**Test**
Suppl. Fig. 4e	*min equivalents*	*Extract of breeding water*	*Flowering rice extract (MR1)*
	0	4902	4669
	16	4594	5736
	32	3466	5915
	48	3438	6143
	64	3491	6826
	80	3497	6825
Suppl. Fig. 4f	*min equivalents*	*Extract of breeding water*	*Flowering rice extract (MR3)*
	0	4902	4669
	16	4400	5473
	32	3746	5978
	48	3687	6427
	64	3731	6325
	80	3185	6700

Supplementary Table 5: Number of gravid *Anopheles arabiensis* and eggs counted in the attraction and oviposition assays in response to the pooled rice headspace extracts of cultivars MR1 and MR3.

Attraction response				Oviposition response			
**Figure**	**Dose**	**Control**	**Test**	**Figure**	**Dose**	**Control**	**Test**
Fig. 1c	*min equivalents*	*Hexane*	*Pooled rice extract (MR1)*	***Fig. 1d***	*min equivalents*	*Hexane*	*Pooled rice extract (MR1)*
	0	15	15		0	3738	3811
	16	14	24		16	3975	6106
	32	18	39		32	3979	6522
	48	19	52		48	3448	5888
	64	17	49		64	2831	6129
	80	11	54		80	2604	7248
Fig. 1e	*min equivalents*	*Hexane*	*Pooled rice extract (MR3)*	***Fig. 1f***	*min equivalents*	*Hexane*	*Pooled rice extract (MR3)*
	0	15	15		0	3738	3811
	16	14	23		16	4077	5125
	32	22	39		32	3726	6131
	48	20	35		48	3272	5883
	64	17	52		64	2688	6280
	80	16	49		80	2884	6988
Fig. 1g	*min equivalents*	*Pooled rice extract (MR1)*	*Pooled rice extract (MR3)*	***Fig. 1h***	*min equivalents*	*Pooled rice extract (MR1)*	*Pooled rice extract (MR3)*
	16	20	29		16	4030	5581
	32	24	43		32	3947	5772
	48	23	45		48	3265	6080
	64	26	56		64	3014	6755
	80	14	61		80	2578	5955
Fig. 1k	*log (dose(ng))*	*Full synthetic blend*	*Pentane*	***Fig. 1l***	*log (dose(ng))*	*Full synthetic blend*	*Pentane*
	0.1	26	24		0.1	4435	4297
	0.3	40	21		0.3	4916	3705
	1	59	19		1	5174	3324
	3	38	17		3	5447	2759
	10	36	16		10	4752	2941
	30	18	27		30	4354	4810
	100	9	20		100	2352	5569

Supplementary Table 6: Number of gravid *Anopheles arabiensis* responding in the attraction assay to the extracts of pooled rice cultivars MR1 and MR3.

	Attraction response		
**Figure**	**Dose**	**Control**	**Test**
Suppl. Fig. 5a	*min equivalents*	*Extract of breeding water*	*Pooled rice extract (MR1)*
	0	21	21
	16	25	32
	32	17	28
	48	21	43
	64	21	53
	80	18	56
Suppl. Fig. 5b	*min equivalents*	*Extract of breeding water*	*Pooled rice extract (MR3)*
	0	21	21
	16	22	36
	32	21	44
	48	21	45
	64	18	59
	80	15	53

Supplementary Table 7: Number of gravid *Anopheles arabiensis* responding in the oviposition assay to the headspace extracts of pooled rice cultivars MR1 and MR3.

	Oviposition response		
**Figure**	**Dose**	**Control**	**Test**
Suppl. Fig. 6a	*min equivalents*	*Extract of breeding water*	*Pooled rice extract (MR1)*
	0	5532	5051
	16	4556	5177
	32	3877	5802
	48	3792	5802
	64	3696	6353
	80	3044	6298
Suppl. Fig. 6b		*Extract of breeding water*	*Pooled rice extract (MR3)*
	0	5532	5051
	16	4415	5371
	32	4366	6198
	48	4141	6489
	64	3831	6451
	80	3557	6182
Suppl. Fig. 6c		*Pooled rice extract (MR1)*	*Pooled rice extract (MR3)*
	16	4132	4751
	32	3849	5300
	48	3458	6032
	64	3421	6534
	80	3301	6311

Supplementary Table 8: Number of gravid *Anopheles arabiensis* responding in the attraction assay to synthetic full and subtractive blends.

Attraction response			
Figure	*Synthetic blend*	*Control (Pentane)*	*Test (synthetic blend)*
**Suppl. Fig. 8**	Full blend	19	59
	Reduced α-pinene	14	30
	Reduced β-pinene	18	42
	Reduced 3-carene	16	31
	Reduced limonene	14	39
	Reduced sulcatone	14	38
	Reduced nonanal	28	28
	Reduced decanal	19	45
	Reduced β-caryophyllene	14	29
	Only nonanal	26	35

Supplementary Table 9: Number of eggs laid by gravid *Anopheles arabiensis* in the oviposition assay to the full and subtractive synthetic blends.

**Oviposition response**			
Figures	*Synthetic blend*	*Control (Pentane)*	*Test (synthetic blend)*
**Suppl. Fig. 9**	Full blend	3324	6474
	Reduced α-pinene	5027	3996
	Reduced β-pinene	5595	3828
	Reduced 3-carene	5054	3758
	Reduced limonene	5268	3778
	Reduced sulcatone	5260	3969
	Reduced nonanal	4335	5107
	Reduced decanal	5296	3807
	Reduced β-caryophyllene	5210	3872
	Only nonanal	4667	3869
